# Effects of Invariant NKT Cells on Parasite Infections and Hygiene Hypothesis

**DOI:** 10.1155/2016/2395645

**Published:** 2016-08-03

**Authors:** Jun-Qi Yang, Yonghua Zhou, Ram Raj Singh

**Affiliations:** ^1^Key Laboratory of National Health and Family Planning Commission on Parasitic Disease Control and Prevention, Jiangsu Provincial Key Laboratory on Parasite and Vector Control, Jiangsu Institute of Parasitic Diseases, Wuxi, Jiangsu 214064, China; ^2^Autoimmunity and Tolerance Laboratory, Division of Rheumatology, Department of Medicine, David Geffen School of Medicine at University of California, Los Angeles, Los Angeles, CA 90095, USA

## Abstract

Invariant natural killer T (iNKT) cells are unique subset of innate-like T cells recognizing glycolipids. iNKT cells can rapidly produce copious amounts of cytokines upon antigen stimulation and exert potent immunomodulatory activities for a wide variety of immune responses and diseases. We have revealed the regulatory effect of iNKT cells on autoimmunity with a serial of publications. On the other hand, the role of iNKT cells in parasitic infections, especially in recently attractive topic “hygiene hypothesis,” has not been clearly defined yet. Bacterial and parasitic cell wall is a cellular structure highly enriched in a variety of glycolipids and lipoproteins, some of which may serve as natural ligands of iNKT cells. In this review, we mainly summarized the recent findings on the roles and underlying mechanisms of iNKT cells in parasite infections and their cross-talk with Th1, Th2, Th17, Treg, and innate lymphoid cells. In most cases, iNKT cells exert regulatory or direct cytotoxic roles to protect hosts against parasite infections. We put particular emphasis as well on the identification of the natural ligands from parasites and the involvement of iNKT cells in the hygiene hypothesis.

## 1. Introduction

Natural killer T (NKT) cells are recently discovered innate-like subset of lymphocytes expressing both NK and T cell markers. NKT cells are a phenotypically and functionally diverse subset of T cells that recognize self- and microbial lipids [1, 2]. Most NKT cells are restricted by MHC-I like molecule CD1, which can further distributed into two major subsets: type I and type II NKT cells (Table 1). Type I NKT cells are also called invariant NKT (iNKT), expressing exclusively limited T cell receptor *α* (TCR*α*) and TCR*β* receptors, that is, V*α*14-J*α*18 predominately paired with V*β*8.2, V*β*7, or V*β*2 in mice and V*α*24-J*α*18 paired with V*β*11 in human [2, 3]. iNKT cells secrete a wide array of cytokines and chemokines immediately upon activation through TCR engagement by glycolipids. iNKT cells exert their regulatory and cytotoxic activities also through direct contact, granzyme B, or FasL-induced mechanisms [4–6]. Hence, iNKT cells exert both regulator and effector cell function and bridge the innate and adaptive immune responses [7]. Recently, iNKT cells have been further classified into NKT1, NKT2, and NKT17 lineages based on their cytokine profiles and distinct transcription factors, T-Bet, Gata-3, and Ror*γ*t, as conventional T helper 1 (Th1), Th2, and Th17 cells [8, 9]. On the other hand, type II NKT cells, which are also called non-iNKT or variant NKT (vNKT) cells, express more diverse TCR*α* and TCR*β* receptors [10]. There still exists a minor group of CD1 nonrestricted NKT cells, referred to as NKT-like cells [11, 12]. The functions of vNKT and NKT-like cells are relatively unknown.

**Figure 1:**
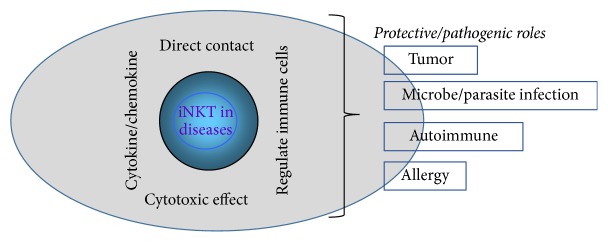
Diagram of iNKT cells in diseases. Activated iNKT cells can secrete a broad range of Th1, Th2, and Th17 effector cytokines and change the cytokine profiles of hosts in vivo. iNKT cells can directly bind target cells, such as autoreactive B cells and pathogens, to mediate cytotoxic activity. Moreover, iNKT cells may cross talk and regulate different T cell subsets. Through these and other mechanisms, iNKT cells exert, in most cases, protective, or otherwise, pathogenic roles in the pathogenesis of various diseases.

A hallmark of iNKT cells is their capacity to rapidly produce copious amounts of cytokines and chemokines upon TCR stimulation, which endows these cells with potent immunomodulatory activities for a wide variety of immune responses and diseases (Figure 1). iNKT cells exhibit potent effector functions and play critical roles in antimicrobial defense, cancer immunosurveillance, and modulation of immune-mediated disorders [13–16]. As iNKT cells recognize glycolipids that are enriched in microbes and parasites, they are believed to play important roles in the infections caused by these pathogens. Recent findings indicate that iNKT cells might be a key player in “hygiene hypothesis,” which tries to explain the declined infections with rising autoimmune and atopic diseases in the recent two to three decades [17, 18]. In this review, we summarize the roles of iNKT cells in parasitic infections, with particular emphasis on the involvement of iNKT cells in the hygiene hypothesis and underlying mechanisms.

## 2. NKT Cells in Parasitic Infections

Albeit being widely studied during viral and bacterial infections, the role of iNKT cells during parasite infections remains largely unexplored. As helminth infections can usually induce Th2-dominated immune responses and iNKT cells can rapidly produce copious amounts of various cytokines including interleukin-4 (IL-4), these cells might be important players in the initial steps leading to Th2 responses during helminthiasis [19]. Recent reports have indicated that NKT cells are involved in the pathogenesis of several parasite infections in animal models and patients, playing, in most cases, protective or regulatory roles towards hosts.

### 2.1. NKT Cells in Helminth Infections

Schistosomiasis remains a severe public health problem in many developing countries in endemic areas. It is caused by digenetic blood trematodes, of which there are three main species:* Schistosoma mansoni*,* S. japonicum*, and* S. haematobium*. Previous studies by others and us indicated that egg deposit in the liver was a determining factor to drive Th2 response in* S. mansoni *and* S. japonicum *infections in mice [20, 21]. NKT cells may contribute to regulating the cytokine secretion profiles during infections. In mice infected with* S. mansoni*, iNKT cells exhibited an activated phenotype. Hepatic iNKT cells produced both interferon-*γ* (IFN-*γ*) and IL-4 following schistosome egg deposited in the liver [22]. Further studies revealed that* S. mansoni* activated both iNKT and non-iNKT cells in vivo. iNKT cells contributed to Th1 cell differentiation, whereas non-iNKT cells might be mostly implicated in Th2 cell differentiation in response to this parasite [23]. Luo and colleagues reported that NK and NKT cells were activated and expanded from draining mesenteric lymph node (MLN) in mice 5–7 wk after infection with* S. japonicum*. These cells produce IL-4 and IL-17 [24]. However, the kinetics of NKT cells and their precise roles in immunomodulation during schistosome infections remain unclear. This is also true to nematode infections, where NKT cells remains ill-defined in their pathogenesis [19]. A pioneer work from Balmer and colleagues found an expansion of NKT (CD3^+^NK1.1^+^) as early as 24 hours following the infection with* Brugia pahangi* [25]. However, depletion of NK1.1-expressing cell had no effect on the Th2 development during the gastrointestinal nematode* Trichuris muris* infection [26].

### 2.2. NKT Cells in Protozoan Infections

iNKT cells have been reported playing crucial roles in the pathogenesis of protozoan infections. In* malaria*, early interactions between blood-stage* Plasmodium* parasites and cells of the innate immune system, including innate-like NKT cells, are important in the timely control of parasite replication and in the subsequent elimination and resolution of the infection [27]. The lipid extracts from murine malaria parasites could actually be loaded onto CD1 molecules to stimulate iNKT cell by the use of artificial antigen-presenting beads [28]. The level of protective antimalaria immunity was greatly enhanced by coadministration of *α*-galactosylceramide (*α*-GalCer) with suboptimal doses of irradiated sporozoites or recombinant viruses expressing a malaria antigen in mice [29]. iNKT cells were increased in numbers and played critical roles through the secretion of IFN-*γ* in reducing liver-stage burden to a secondary infection by murine malaria* Plasmodium yoelii* [30]. *α*-C-GalCer displayed a superior inhibitory activity against the liver stages of the rodent malaria parasite* P. yoelii* compared to its parental glycolipid, *α*-GalCer [31]. *α*-GalCer and its analogs have also been used as adjuvants for malaria vaccine. This adjuvant effect depended on NKT cell activation, which was able to boost IFN-*γ* production by NK cells and memory CD8^+^ T cells [32].

*Visceral leishmaniasis* (Kala-azar) is a deadly disease caused by the parasitic protozoa* Leishmania donovani*. iNKT cells are involved in the pathogenesis of leishmaniasis. In patients with visceral leishmaniasis, bone-marrow-derived non-iNKT cells dominantly produced IFN-*γ* in response to* L. donovani* antigen in vitro [33]. Post-kala-azar dermal leishmaniasis is a chronic dermal complication that occurs usually after recovery from visceral leishmaniasis. There was a raised proportion of circulating NKT cells in these patients compared to health controls [34]. Karmakar and colleagues isolated a natural ligand of NKT cells, *β*-(1–4)-galactose terminal glycosphingophospholipid (GSPL) from this parasite to treat infected BALB/c mice. This immunotherapy with GSPL induced IFN-*γ* through the cooperative action of TLR4 and NKT cells, which contributed to the effective control of acute parasite burden in the infected animals [35]. By use of iNKT cell-deficient (J*α*18^−/−^) C57BL/6 mice, another study demonstrated that iNKT cells played a role in early and sustained proinflammatory cytokine response warranting efficient organization of hepatic granulomas and parasite clearance of* L. donovani* [36]. NKT cell activation by *α*-GalCer during intradermal DNAp36 priming was highly protective against murine cutaneous leishmaniasis, resulting in the heightened activation and development of CD4^+^ and CD8^+^ effector and memory T cells [37]. Conversely, activation of iNKT cells exacerbated, rather than ameliorated, experimental visceral leishmaniasis by* L. donovani*, which was correlated with a bias towards increased IL-4 production by iNKT [38]. This may illustrate the double-edged sword of NKT cell-based therapy in leishmaniasis.

iNKT cells protect mice against* Toxoplasma gondii* infection. By oral infection of mildly virulent strain ME49* T. gondii* cysts, most CD1d-deficient C57BL/6 mice died within 2 wk of infection compared to no death in WT mice [39]. After activation with* T. gondii*, NKT cells were important mediators of the immune response via a robust IFN-*γ*-mediated effect that limited parasite replication and allowed for parasite clearance [40]. Nevertheless, this strong Th1 response, when uncontrolled, can mediate the lethal ileitis. Treatment of mice with a single injection of *α*-GalCer one day before infection activated intestinal NKT and led to a shift in cytokine secretion towards a Th2 profile and a dramatic increase in Treg cells in MLNs, which alleviated intestinal lesions and increased survival of mice [40]. On the other hand, iNKT cells may negatively regulate the immune response against* T. gondii* infection possibly by producing IL-4 and suppressing the induction of heat shock protein 65. The latter is induced in host macrophages by *γδ*T cells and plays an essential role in protective immunity in this infection [41].

NKT cells are involved in the pathogenesis of some* other protozoan infections*, providing protection against infections in most cases. CD8^+^ NKT cells were able to activate macrophages to kill* Trypanosoma congolense* through the production of nitrogen oxides, whereas Treg cells prevented the activation of the CD8^+^ NKT cells [42]. However, another report indicated that loss of iNKT cells did not affect the susceptibility or resistance in CD1d^−/−^ C57BL/6 mice to the infections with virulent African trypanosomes,* T. congolense* or* T. bruce* [43]. Lotter and colleagues identified a lipopeptidophosphoglycan from* Entamoeba histolytica* membranes (EhLPPG) as a possible iNKT natural ligand. EhLPPG treatment, similar to *α*-GalCer application, induced protective IFN-*γ* but not IL-4 production from iNKT cells and significantly reduced the severity of amebic liver abscess in mice infected with* E. histolytica* [44]. By the use of CD1d KO mice, it was found that iNKT cells contributed to resistance against this protozoan and to the control of inflammation in the colitis induced by the infection [45]. iNKT cells play important roles in the pathogenesis of some other parasitic diseases, as well as of a wide range of microbe infections, as seen in recent nice reviews [10, 46, 47].

### 2.3. Underlying Mechanisms

NKT cells play protective role against a wide range of parasite infections as discussed above, whereas the underlying molecular mechanisms are not fully elucidated. Shifting of host's cytokine secretion profiles may account for the protective or, in some cases, pathogenic effects of NKT cells on parasitic infections. Activated iNKT cells can also transactivate many other immune cells or attract these cells to the sites of infection to exert their regulatory roles. Like NK cells, activated NKT cells can also mediate cytotoxic activity, possibly involving both perforin/granzyme and Fas/FasL pathways [5, 6, 48]. This function could be relevant to immunity against intracellular microorganisms and tumors [49]. Parasites are enriched in lipid, which may contain natural ligands for NKT cells as discussed in the next section. Therefore, it is not surprising that iNKT cells participate in the pathogenesis of a range of different parasitic infections. Further detailed studies are needed before developing iNKT-based therapy to parasite infections. The role of iNKT cells in some parasitic infections and possible effect mechanisms are summarized in Table 2.

## 3. Contribution of NKT Cells to Hygiene Hypothesis

### 3.1. Hygiene Hypothesis

The “hygiene hypothesis” was proposed in 1989 by Strachan [17] to explain the dramatic increase in the prevalence of autoimmune and allergic diseases over the past two to three decades [18]. According to this hypothesis, reduced exposure to microorganisms and parasites in childhood is the main cause for the increased incidence of both Th1-mediated autoimmune diseases and Th2-mediated allergic diseases. Currently, the hypothesis is becoming more accepted with accumulating epidemiological and clinical evidences to support [50–52]. Although the exact scientific underpinnings for the hygiene hypothesis and the underlying mechanisms by which infections affect the immune system to prevent diseases have remained a puzzle over the years for both scientists and clinicians [18], there is increasing recognition that exposure to infectious agents evokes fundamental effects on the development and behavior of the immune system [18, 53]. The core of this hypothesis consists in the notion that the microbial environment interfaces with the innate immune system and modulates its ability to impart instructions to adaptive immune responses, particularly when such interactions occur in utero and/or in early life [54]. Many chronic infections, both microbial and parasitic, induce forms of immune suppression or downmodulation [55]. Recent studies have indicated that infectious agents stimulate a large variety of regulatory T cells, such as Th2, Treg, Tr1, and NKT cells, which secrete immunosuppressive cytokines/chemokines, such as IL-10 and TGF-*β* to alter the Th1/Th2 balance. Additionally, the innate immune system is also associated with the hygiene hypothesis. Infections may induce the generation of regulatory macrophages, dendritic cells, innate lymphoid cells (ILC), NK, and B cells [56–58]. TLR-MyD88 pathway is believed to be involved in the induction of different subsets of regulatory T cells, such as Treg and NKT cells [50]. The ability of infectious agents to regulate the immune system of their host is an increasingly fascinating topic [53].

### 3.2. Parasites and Hygiene Hypothesis

Helminths, as long-lived parasites, are remarkable for their ability to manipulate host immunity, protecting themselves from elimination and minimizing severe pathology in the host [53, 56, 59, 60]. Immunomodulation by parasitic helminths is a general phenomenon that is conserved across species, classes, and even phyla [61]. Therefore, parasitic infections are a major theme in the hygiene hypothesis. Allergies and autoimmune diseases are less prevalent in countries with higher burdens of helminths and other parasitic organisms [55]. There are strong epidemiological evidences to support the premise that the dramatic increase in atopic disease in the developed world is a direct consequence of the eradication of helminth infections [58]. At least some helminthes seem to have antiallergic or anti-inflammatory effects in humans. Experimental evidences have also shown the significant suppression for the development of airway hyperresponsiveness (AHR) in mice infected with numerous helminths, including blood fluke* Schistosoma japonicum* [62], filaria* Litomosoides sigmodontis* [63], nematode* Heligmosomoides polygyrus* [64], and* Nippostrongylus brasiliensis* [65]. These mice show attenuated airway inflammation with reduced infiltration of eosinophilia in the BAL and lung and allergen-specific IgE in sera. Many studies have also demonstrated that helminth infections lower the risk of autoimmunity. Experimental studies have also shown protective effects of helminth infections in animal models of autoimmunity. Surprisingly, helminths have been shown to suppress various types of autoimmune disease, such as collagen-induced arthritis, experimental autoimmune encephalomyelitis, and type 1 diabetes in murine models as reviewed recently [51]. Helminth infections might be beneficial to the induction of multiple regulatory mechanisms, including various regulatory cell populations, inhibitory receptors, blocking antibodies, and two prominent cytokines: IL-10 and TGF-*β* [61, 66]. Thus, it is not surprising that helminths can modulate immunopathology, whether in the context of allergic inflammation or autoimmune disease, either directly or indirectly [55].

Infections by protozoan, like helminth, also modulate host immune system. A study shows that serum antibodies to* Toxoplasma gondii* tend to be negatively associated with allergic sensitization to food and aeroallergens in children from different geographical areas in Greece, Netherlands, China, India, and Russia [67]. A negative association also exists between* T. gondii* infection and the presence of multiple sclerosis [68].

### 3.3. iNKT Cells in Hygiene Hypothesis

Nevertheless, the roles of iNKT cells in the hygiene hypothesis mostly remain unknown. The literature is limited regarding the involvement of iNKT cells in this hypothesis. On the other hand, many microorganisms and parasites contain various lipids in their structures. The bacterial and parasitic cell wall is a cellular structure highly enriched in a variety of glycolipids and lipoproteins [49]. Along with *α*-GalCer, growing evidences suggest that some microorganisms, including* Mycobacteria*,* Sphingomonas*,* Borrelia*,* Helicobacter pylori*,* Streptococcus pneumoniae*, and* Group B streptococcus*, can produce CD1d-restricted ligands capable of activating a proportion of iNKT cells [2]. Hence, iNKT cells can respond directly by recognizing the glycolipid antigens expressed by these bacteria. iNKT cells can also respond indirectly to many other bacteria such as* Salmonella enterica* and* Staphylococcus aureus* [18]. Of note, some protozoan and helminthic parasites also contain natural ligands of NKT cells and several candidates have been successfully isolated. The excretory and secretory (ES) products, which are often glycosylated, are found in the bloodstream of infected hosts and dictate particular functional immune responses that allow persistence of the parasite, typically by inducing Th2-associated cytokines and expansion of various regulatory cell subsets, including NKT cells [22, 56, 69]. The adult worms of* Schistosoma mansoni* express a range of glycoconjugates, such as galactosylceramide and glucosylceramide [70], which may contain natural ligands of NKT cells. Infection with* S. mansoni* or exposure to eggs from this helminth inhibited the development of type 1 diabetes in NOD mice [71]. In addition, soluble extracts from worms or eggs of this schistosome possess the similar ability as infection to prevent the onset of diabetes if injection is given at early age (4 wk old). Soluble adult worm antigen or soluble egg antigen may expand the iNKT cell population in NOD mice, although the lipids binding to iNKT cells have not been targeted in this study [71]. Lipid extracts from murine malaria parasites can actually be loaded onto CD1 molecules to expand iNKT cells [28]. Lotter and colleagues have identified a lipopeptidophosphoglycan from* E. histolytica* membranes as a possible iNKT natural ligand, which can stimulate iNKT cells to produce IFN-*γ* to exert protective role against this infection [44]. Karmakar and colleagues isolated a natural ligand of NKT cells, *β*-(1–4)-galactose terminal glycosphingophospholipid (GSPL), from* L. donovani* to treat infected BALB/c mice [35]. These investigations pave the way to identify natural ligands of iNKT cells for the development of novel therapeutic agents.

iNKT cells may exert their immunomodulatory effects in hygiene hypothesis through the interaction with Treg, Th17, and other immune cells. Upon activation with microbial and parasitic lipid antigens, iNKT cells rapidly produce a wide range of cytokines and chemokines, transactivating many immune cell types, such as Th1, Th2, and Treg. In EAE model, iNKT cells were found necessary for maintaining the mesenteric Th17 cells. Th17 cells in the MLNs are greatly reduced in* CD1* ^*−/−*^ mice or* Jα281* ^*− /−*^ mice [72], which lack iNKT cells. iNKT cells induce the conversion of naïve diabetogenic BDC2.5 T cells into Foxp3(+) Treg cells in the pancreatic lymph nodes accumulating in the pancreatic islets [73]. In addition, iNKT cells can suppress both antigen-induced acute arthritis and collagen-induced chronic arthritis, likely via inhibition of arthritogenic Th1 cells [74]. In* Toxoplasma gondii* infection, activation of iNKT cells by *α*-GalCer can lead to a shift to Th2 cytokine profile and a significant increase in Treg cells in MLNs, which exerts protective role and increases survival of mice [40]. The recently defined innate lymphoid cells (ILC) [75] share some features with iNKT cells. Upon activation without the need of prior sensitization, both cells can release copious amounts of Th1, Th2, and/or Th17 cytokines that shape subsequent innate and adaptive immune responses [76]. Although sparse up to now, there exists experimental evidence for direct interactions of ILCs and NKT cells possibly via their effector cytokines [77]. For example, NKT cells, as well as alveolar macrophages, secrete endogenous IL-33 that enhance IL-5 production from ILC2 in lungs during influenza virus infection [78]. The interaction of iNKT cells with other T cell subsets and underlying mechanisms remain to be elucidated.

**Figure 2:**
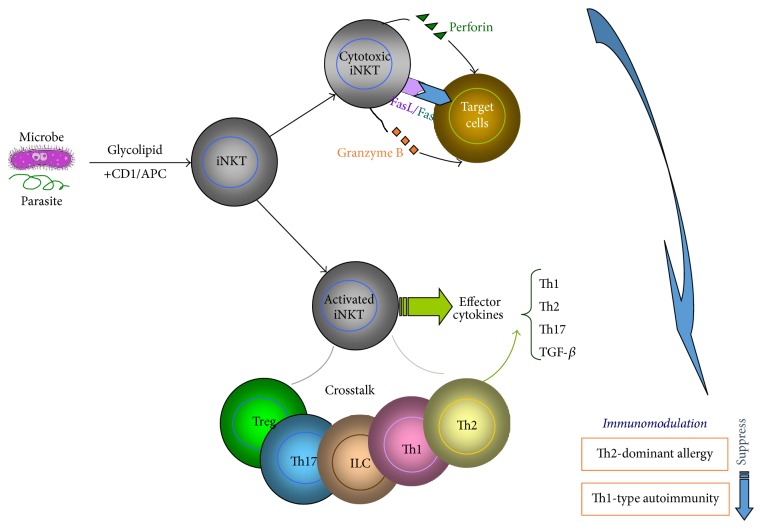
Diagram of iNKT cells in hygiene hypothesis. iNKT cells can be activated by glycolipids from microbes and parasites, which bind to CD1 molecules on antigen-presenting cell (APC). Activated iNKT cells secrete a broad range of effector cytokines. They also mediate cytotoxic activity, possibly involving both perforin/granzyme B and Fas/FasL pathways. Moreover, iNKT cells may cross talk with Treg, Th17, ILC, and Th1/2 subsets via secreted cytokines or direct contact. iNKT cells may exert their immunomodulatory effects in hygiene hypothesis through the above combined and other effects.

Taken together, microbe and parasite infections, especially at early lifetime, may sincerely modulate the host's immune system. Parasitic worms are able to survive in their mammalian host for many years due to their ability to manipulate the immune response. The underlying mechanisms regarding how infections affect the immunity of hosts remain to be clarified. Upregulation of regulatory T cell subsets, such as Treg, and induction of inhibitory cytokines and/or chemokines are the common findings. The involvement of NKT cells in the hygiene hypothesis mostly remains elusive. Given the fact that many microbes and parasites are enriched in lipid antigens and NKT cells are unique T cell subset that can recognize lipid antigens, it is reasonable to speculate that NKT cells play key roles in this hypothesis (Figure 2). Further studies are needed to verify this idea.

## 4. Conclusions

iNKT cells are unique innate-like T cell subset that bridge between innate and acquired immunity systems. iNKT cells exert both effector and regulatory functions through direct contact or quick secretion of copious amounts of cytokines, chemokines, and other mediators upon their TCR engagement by glycolipid antigens. These cytokines and chemokines critically regulate the downstream differentiation of Th1, Th2, Th17, and other cells. Therefore, iNKT cells have been postulated to have an important proximal immunoregulatory role and influence both innate and acquired immune systems. iNKT cells play crucial regulatory roles in autoimmunity, allergy, and infections. They participate in the host's immunity and immunopathogenesis of a wide range of parasite infections as discussed above. More thorough investigation is clearly necessary to better define their mode of activation and their regulatory functions in parasitic infections. Although the involvement of iNKT cells in the hygiene hypothesis and the contribution to autoimmunity and allergic inflammation remains to be fully elucidated, exploiting iNKT cells in helminth immunomodulatory mechanisms may lead to opening a new avenue to develop novel safer therapeutic agents for these diseases based on the manipulation of iNKT cell function.

## Figures and Tables

**Table 1:** Classifications of NKT cells.

	Type I	Type II	NKT-like
Alternative name	iNKT	Non-iNKT, vNKT	
CD1-dependent	Yes	Yes	No
TCR*α*-chain	V*α*14-J*α*18 (mice)	Diverse	Diverse
	V*α*24-J*α*18 (humans)		
TCR*β*-chain	V*β*8.2, V*β*7, and V*β*2 (mice)	Diverse	Diverse
	V*β*11 (human)		
*α*-GalCer response	Yes	No	No
Cytokine profile	Th1, Th2, and Th17	Th1, Th2	Th1

**Table 2:** Summary of NKT cells on parasite infections.

Parasites	Host	Model or treatment	Effect-mechanism	NKT overall function	Ref.
*Schistosoma mansoni*	C57BL/6	CD1 KO	IL-4 ↑ IFN-*γ* ↑	Activated	[22, 23]
*Schistosoma japonicum*	C57BL/6	WT	IL-4 ↑ IL-17 ↑	Activated	[24]
*Brugia pahangi*	C57BL/6	WT	IL-4 ↑	Activated	[25]
*Trichuris muris*	B10.BR	NKT deletion	IL-4~	Protective	[26]
*Plasmodium yoelii*	BALB/c C57BL/6	CD1 KO	IFN-*γ* ↑	Protective	[27]
*Leishmania donovani*	patient		Non-iNKT →IFN-*γ* ↑	Protective	[33]
*Leishmania donovani*	BALB/c C57BL/6	J*α*18 KO, *α*-GalCer	IL-4 ↑ and/or IFN-*γ* ↑	Protective	[35–37]
*Leishmania donovani*	C57BL/6	*α*-GalCer	IL-4 ↑	Pathogenic	[38]
*Toxoplasma gondii*	BALB/c C57BL/6	CD1 KO, *α*-GalCer	IFN-*γ* ↑	Protective	[39, 40]
*Trypanosoma congolense*	BALB/c C57BL/6	Anti-CD1d, CD1 KO		Protective or suppressive	[42]
				No effect	[43]
*Entamoeba histolytica*	C57BL/6	J*α*18 KO, CD1 KO	IFN-*γ* ↑	Protective	[44, 45]

→: induce; ↑: increase; and ~: no change.
